# Fast drying of Fine Needle Aspiration slides using a hand held fan: impact on turn around time and staining quality

**DOI:** 10.1186/1742-6413-3-12

**Published:** 2006-04-19

**Authors:** Mirza A Baig, Lamia Fathallah, Jining Feng, Mujtaba Husain, David G Grignon, Mousa A Al-Abbadi

**Affiliations:** 1 Department of Pathology, Wayne State University/Detroit Medical Center, Detroit, MI, USA

## Abstract

To analyze the impact of using a hand held fan to speed the air-drying process during immediate adequacy evaluation of Fine Needle Aspirations. The effect on turn around time and staining quality is evaluated.

Two mirror image air-dried smears for each pass were prepared. One was subjected to a small hand-held fan with a fan diameter of 7 cm held an average distance of 3 to 5 cm from the slide. The other smear was left to dry without a fan. A total of 93 consecutive pairs were evaluated over a 2-month duration.

The average time needed for air-drying using the fan was 73 seconds (range 10–300 seconds, standard error 6.986), while it was 200 seconds (range 15–645 seconds, standard error 17.799) for those without fan. This difference was statistically significant (p < 0.001). Smears were then evaluated for single cells, cell clusters and background material and no appreciable difference in stain quality was noted between the 2 groups.

The use of a small hand-held fan for air-drying shortened the drying time for FNA adequacy by an average of 127 seconds (63% time reduction) for each pass. The quality of staining was comparable. Using a fan is highly recommended.

## To the editor

Fine needle aspiration biopsy (FNAB) is a well recognized initial diagnostic approach with high sensitivity, specificity and accuracy [1]. It has been demonstrated, especially in the current era of health care cost consciousness, that FNAB is more cost effective than a tissue biopsy [2]. In addition, the diagnosis can be made in most cases immediately or at the most within 24 hours of the procedure. This makes this approach an attractive option for clinicians that need a quick answer to common diagnostic problems.

The latter objective has been successfully achieved through the use a short list of quick and fast stains that are performed at the time of the procedure [3]. Those stains are collectively characterized by their ease of performance and amenability for performance on site with acceptable quality thus allowing for the immediate interpretation and triage by pathologists. Institutions with an active and busy fine needle aspiration (FNA) service, such as ours, provide the immediate adequacy evaluation and triage at different locations including FNA clinics, radiology suites, out patient clinics, operating rooms and at the bed side as a point-of-care service. The number of these procedures is increasing in frequency and it is highly advantageous to have a pathologist on site performing or providing the immediate evaluation and triaging of the sample for appropriate ancillary studies [4].

The stains to be performed can be either air-dried based smears such as the Diff Quik (DQ) stain or ethanol-fixed type stains such as the ultrafast Papanicolaou stain[5]. The modified Romanowsky stain, also known as Diff Quik (DQ), is used in our laboratory for that purpose. It is cheap, convenient, easy to handle, consistent and quick. The DQ stain is known to be good for highlighting the background matrix material, organisms and the cytoplasmic contents [3]. However, for optimum nuclear detail, the Papanicolaou stain is superior and some institutions utilize the ultrafast method of this stain in addition to or as a replacement for the DQ stain [6]. The DQ stain requires air drying of smears, a step that consumes a variable amount of time and sometimes becomes the rate-limiting step of the procedure. This is usually dependent on the vascularity and consistency of the organ or tissue aspirated. For example, thyroid gland aspirations, a common and frequent FNAB target, are usually bloody and air-drying is frequently time consuming [7]. Reducing the time spent on site by the cytopathology team is an important goal in achieving maximum efficiency. The cost-effective advantages are obvious, particularly in a busy FNAB service [8].

In our institution, we routinely perform immediate adequacy and triage on all in house FNA biopsies. In this prospective study, we evaluated the impact of fast drying using a hand held fan. The effects on time conservation and the quality of the DQ stain are presented.

This study was performed at Wayne State University and the Detroit Medical Center Hospitals. All FNAs performed over a 2-month period in those hospitals that required immediate adequacy evaluation and triage were included in the study.

**Figure 1:**
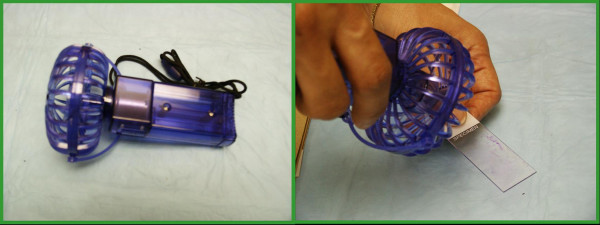
The Fan: blade diameter of 7 cm. and the distance from the slides is 3–5 cm.

Two mirror image air-dried smears for each pass were prepared during the immediate adequacy evaluation. One was air-dried using a hand held fan (Necklace fan, Item # 999338-MMIV, Rite Aid Corporation, Harrisburg, PA) and the other was left to dry without a fan (Figure 1). The diameter of fan blade was 7 cm. The distance from the fan to slide was 3 to 5 cm. The air drying process was judged by visual evaluation of smears for both pairs. This was subjectively judged by looking at the smears until all the areas in the slides appear dried completely. Smears were then evaluated for single cells, cell clusters and background material and no appreciable difference in stain quality was noted between the 2 groups. This evaluation was done by visual assessment by all of us that included blind review of fan-dried and non fan-dried slides. The statistical analysis was performed using SPSS for Windows 12.0 program (SPSS_Inc _Chicago, IL).

**Figure 2:**
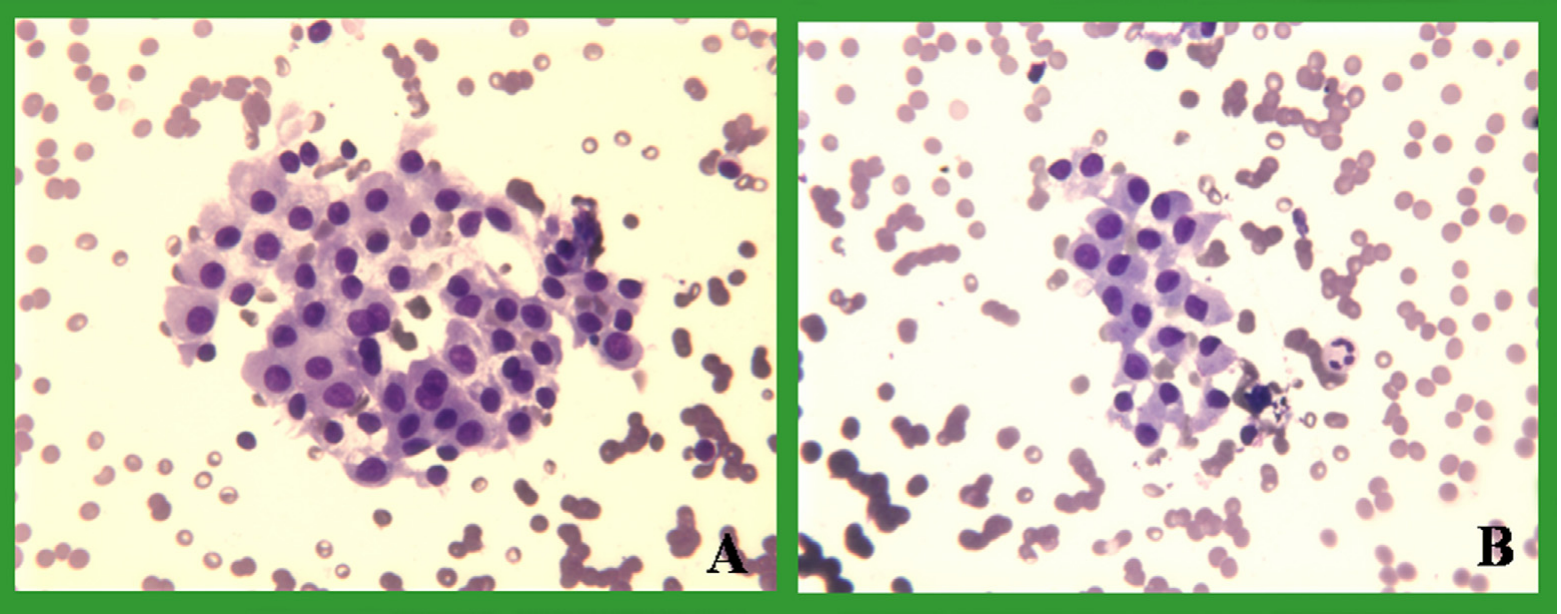
Thyroid FNA: DQ stain of clusters of Hurtle cells from a Hurtle cell neoplasm. A dried with fan & B without a fan.

**Figure 3:**
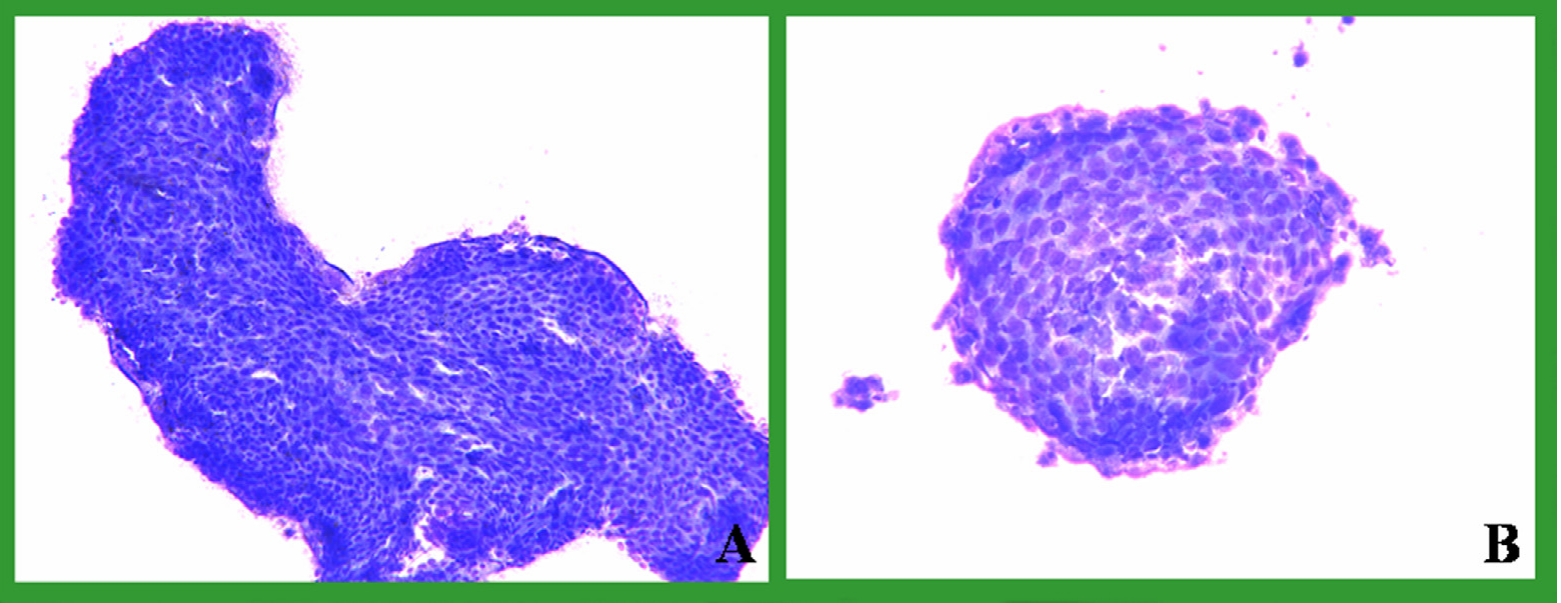
Neck FNA: DQ stains showed clusters of malignant squamous cell. A dried with fan & B without a fan.

**Figure 4:**
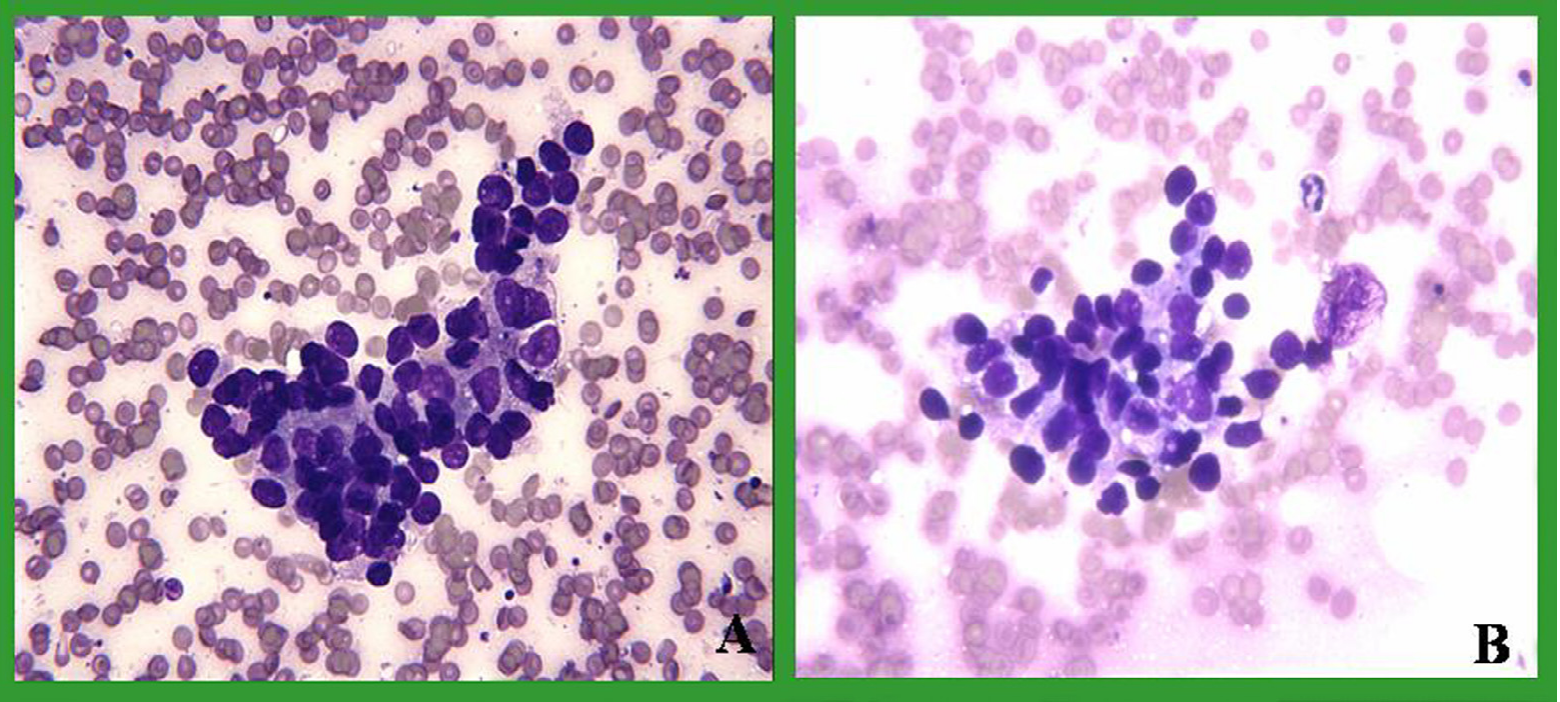
Lung FNA: DQ stains shows clusters of atypical epithelial cells with molding consistent with small cell carcinoma. A dried with fan & B without a fan.

**Figure 5:**
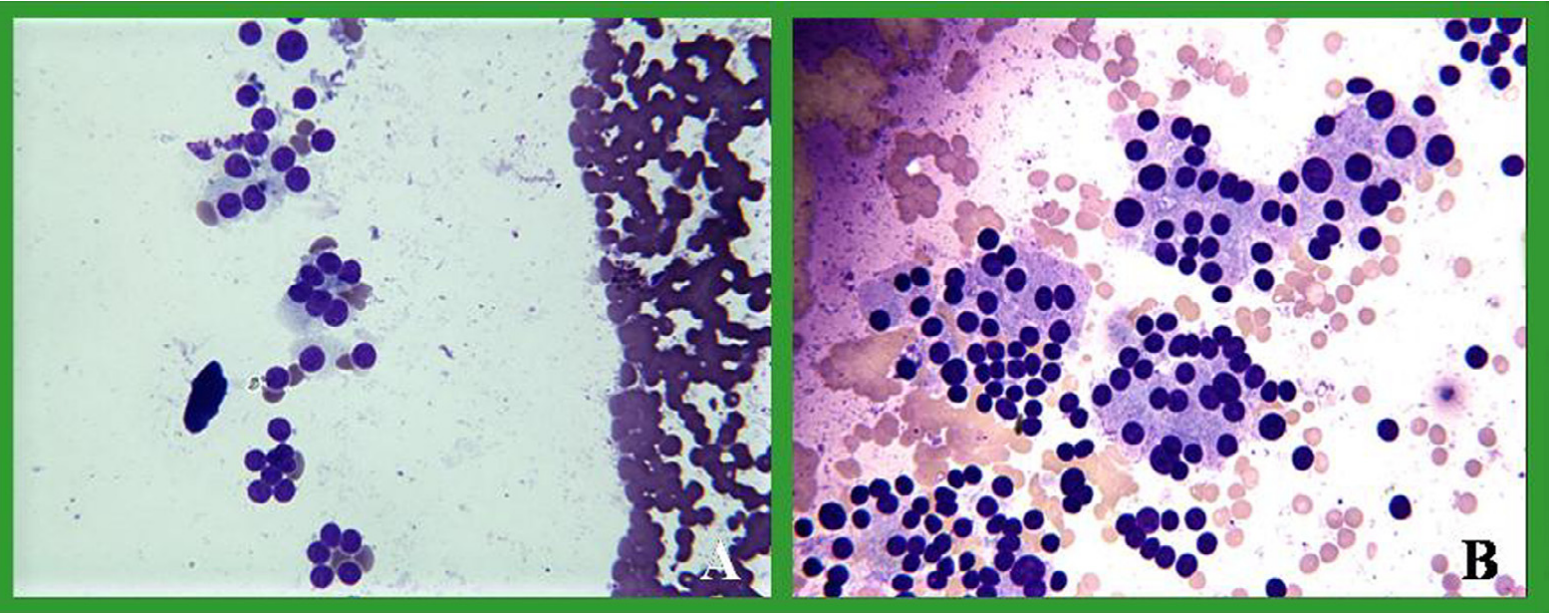
Thyroid FNA: DQ stain of benign clusters of thyroid follicular cells and colloid representing a colloid nodule. A dried with fan & B without a fan.

Slides from 93 pairs were evaluated over a two-month period from 07-15-04 to 09-15-04. The drying time and staining quality of both groups were compared and evaluated. The mean time needed for air-drying using the fan was 73 seconds (range 10–300 seconds, standard error 6.986), and without the fan was 200 seconds (range 15–645 seconds, standard error 17.799) leading to 127 (63%) seconds of shortening. This difference was statistically significant (p < 0.001). Smears were evaluated for single cell, cell clusters and background stain and were found comparable for both groups. No discernible quality difference was present in the Diff-Quik stains between the 2 groups (Figures 2, 3, 4, 5). As shown in those images, the quality of stain appears comparable between the 2 groups. In fact, some of us believe that the quality of the stain improved in certain cases. In addition, blinded review of slides from both groups was performed and revealed no appreciable difference. The way this blinded review was performed is by giving the slides from one group to one of us and to see if he or she can recognize to which group those slides belong, with or without fan. No effects were noted on the number of passes after adopting this method in our institution.

Fine needle aspiration biopsy (FNAB) is currently a standard initial diagnostic procedure that has proven to be accurate and cost effective [1,2,9]. Although arguments were raised regarding the cost of cytopathologists during the on-site evaluation, others including us strongly believe that the availability of immediate triage is still more cost effective [4,9]. Therefore the number of FNA procedures that are performed is increasing in number [10]. Immediate evaluation and a preliminary diagnosis of cytological smears is desirable and in many occasions has a tremendous positive impact on patients, radiologists and the treating clinicians. Taking into consideration all the aforementioned factors, the time that is spent by the pathology team becomes a crucial aspect, not only to speed up the process, but also to decrease the cost that is incurred by the procedure. The medical personnel who are involved in the FNAB procedure and ultimately will be affected by the time saving protocol from this study includes clinicians and their assistants, radiologists and their assistants, cytotechnologists and pathologists. Although it is difficult to measure the exact time spent to perform and triage a complete FNA procedure, we have demonstrated in this study that using the fan reduced the drying time by 70% and consequently will shorten the total procedure time. It is well known there are many variables that dictate the length of procedure time. Those include the number of passes, the number of people involved, the type of stain used and the distance traveled to the procedure place. Therefore, attempting to calculate the exact cost-effective impact in dollars is difficult and varies from institution to institution. However, we believe that shortening drying time and consequently the total procedure time would have a positive impact on the total cost particularly in centers with busy FNA service. Moreover, the FNA operation will be more efficient, since it permits performing more procedures in a given day of operation. As demonstrated in this study, the quality of the smears was comparable between the 2 groups. Therefore, the use of hand-held fan is now routinely used for immediate drying of all cytology smears in our institution. In cases where more than one pair of slides is present, air-drying using the same fan is carried out.

In conclusion, using a hand held fan to speed air-drying time during immediate adequacy evaluation and triage decreased the drying time by 70% without affecting the stain quality of slides. It is believed that using the fan will make the process more efficient and hopefully more cost effective. Use of a fan for air drying is highly recommended.
